# Identification of Two Novel HLA-A*0201-Restricted CTL Epitopes Derived from MAGE-A4

**DOI:** 10.1155/2010/567594

**Published:** 2011-02-14

**Authors:** Zheng-Cai Jia, Bing Ni, Ze-Min Huang, Yi Tian, Jun Tang, Jing-Xue Wang, Xiao-Lan Fu, Yu-Zhang Wu

**Affiliations:** ^1^Department of Immunology, Third Military Medical University, Chongqing 400038, China; ^2^Department of Dermatology, Southwest Hospital, Third Military Medical University, Chongqing 400038, China

## Abstract

MAGE-A antigens belong to cancer/testis (CT) antigens that are expressed in tumors but not in normal tissues except testis and placenta. MAGE-A antigens and their epitope peptides have been used in tumor immunotherapy trials. MAGE-A4 antigen is extensively expressed in various histological types of tumors, so it represents an attractive target for tumor immunotherapy. In this study, we predicted HLA-A*0201-restricted cytotoxic T lymphocyte (CTL) epitopes of MAGE-A4, followed by peptide/HLA-A*0201 affinity and complex stability assays. Of selected four peptides (designated P1, P2, P3, and P4), P1 (MAGE-A4_286-294_, KVLEHVVRV) and P3 (MAGE-A4_272-280_, FLWGPRALA) could elicit peptide-specific CTLs both *in vitro* from HLA-A*0201-positive PBMCs and in HLA-A*0201/K^b^ transgenic mice. And the induced CTLs could lyse target cells in an HLA-A*0201-restricted fashion, demonstrating that the two peptides are HLA-A*0201-restricted CTL epitopes and could serve as targets for therapeutic antitumoral vaccination.

## 1. Introduction

The melanoma antigen genes family A (*MAGE-A*) consists of 12 closely related genes (*MAGE-A1 *to* A12*) located in the q28 region of chromosome X [1, 2]. *MAGE-A*-encoded antigens (MAGE-A) belong to cancer/testis (CT) antigens. These antigens are expressed in various histological types of carcinomas, but not in normal tissues with the exception of testis and placenta [3–8]. Although testis expresses MAGE-A antigens and placenta also expresses some of them [9], testis and placenta do not express MHC class I molecules and therefore cannot be attacked by cytotoxic T lymphocytes (CTLs) specific for these antigens. Thus, MAGE-A antigens are appealing targets for antitumor immunotherapy. A number of clinical trials of therapeutic vaccination have been performed, based on these antigens and their epitope peptides. In some clinical trials executed with short peptides, tumor regressions have been observed in a minority of patients [10–12].

Of the *MAGE-A* family, *MAGE-A4* is one of genes that are abundantly expressed by many tumors of different histological types, such as urothelial carcinoma, bladder cancer, lung cancer, ovarian neoplasm, esophageal squamous cell carcinoma, and oral squamous cell carcinoma [4, 13–17]. Up to now, at least four variants have been found for this gene. The four variants encode the same protein referred to MAGE-A4. MAGE-A4 is found to interact with the liver oncoprotein gankyrin and suppress the tumorigenic activity of gankyrin [18]. Its carboxyl-terminal fragment of 107 amino acids induces p53-dependent and p53-independent apoptosis in human cells [19]. Moreover, the expression of MAGE-A4 may increase caspase-3 activity and promote tumor cell death [14].

Recent studies have identified several antigenic peptides presented by HLA class I molecules, including HLA-A24 restricted MAGE-A4_143–151_ (NYKRCFPVI) peptide, HLA-A1 restricted MAGE-A4_169–177_ (EVDPASNTY) peptide, HLA-B37 restricted MAGE-A4_156–163_ (SESLKMIF) peptide, and HLA-A*0201 restricted MAGE-A4_230–239_ (GVYDGREHTV) peptide [20–23]. It is reported that a polyepitope vaccine targeted to one antigen may elicit strong antigen-specific CTLs to protect against tumor challenge and almost each epitope in the polyepitope can induce specific CTL immune response [24, 25]. On the other hand, about 50% of Caucasians and Asians express HLA-A*0201 [23]. So the identification of more HLA-A*0201-restricted epitopes for MAGE-A4 is likely to provide alternative candidates for the future of clinical trials with defined antigenic peptides and facilitate the design of antitumor vaccines with high efficacy.

To identify epitopes capable of inducing MAGE-A4-specific HLA-A*0201-restricted CTLs, we first predicted HLA-A*0201-restricted epitopes of MAGE-A4 and measured HLA-A*0201 binding capacity of the candidate epitope peptides. We then induced MAGE-A4-specific CTLs from HLA-A*0201 peripheral blood mononuclear cells (PBMCs) with these candidate peptides to seek CTL epitopes from MAGE-A4 antigen, followed by validation for *in vivo* immunogenicity.

## 2. Material and Methods

### 2.1. Cell Lines and Animals

 The HLA-A*0201-expressing human tumor cells T2 (deficient in TAP1 and TAP2 transporters), MCF-7 (breast cancer; MAGE-A-negative [26]), and BB7.2 hybridoma producing anti-HLA-A2 monoclonal antibody (mAb) were purchased from American Type Culture Collection (ATCC, USA). Human melanoma cells LB1751-MEL expressing MAGE-A and HLA-A*0201 were kindly provided by Dr. F. Brasseur (Ludwig Institute for Cancer Research, Brussels, Belgium).

HLA-A*0201/K^b^ transgenic mice (6–8 weeks old) were purchased from the Jackson Laboratory (USA). Animal experiments were performed in accordance with the guidelines of the Animal Care and Use Committee of Third Military Medical University.

### 2.2. Epitope Prediction and Peptide Synthesis

 The MAGE-A4 protein sequence was analyzed for 9-amino acid long peptides, which could potentially bind to HLA-A*0201 molecule, using the computer-based epitope prediction programs BIMAS () and SYFPEITHI (http://www.syfpeithi.de/Scripts/MHCServer.dll/EpitopePrediction.htm). The selected candidate peptides and control peptides (HBcAg_18–27_ and OVA_257–264_) were synthesized by Fmoc chemistry (Sangon, China) and purified by HPLC to a purity of >95%.

### 2.3. Affinity Measurement of Peptide for HLA-A*0201

 The affinity of peptides for HLA-A*0201 was measured as described previously [27]. Briefly, T2 cells were incubated with various concentrations of each peptide and 3 **μ**g/mL human **β**2m in serum-free RPMI 1640 medium at 37°C for 16 h. Then, the cells were washed and stained with antiHLA-A2 mAb and FITC-labeled goat anti-mouse IgG. The expression of HLA-A*0201 on T2 cells was determined with FACS Calibur flow cytometer (Becton Dickinson, USA). For each peptide concentration, the percent mean fluorescence index (% MFI) increase of HLA-A*0201 molecule was calculated as follows: % MFI increase = [(MFI with the given peptide − MFI without peptide)/(MFI without peptide)] × 100.

### 2.4. Assessment of Peptide/HLA-A*0201 Complex Stability

 As previously described [28], T2 cells (10^6^/mL) were incubated overnight with 100 **μ**M of each peptide in serum-free RPMI 1640 medium supplemented with 100 ng/mL human **β**2m at 37°C. Then, they were washed to remove free peptides and incubated with 10 **μ**g/mL of Brefeldin A (Sigma-Aldrich, USA) for 1 h to block newly synthesized HLA-A*0201 molecules to be expressed on cell surface, washed and incubated at 37°C for 0, 2, 4, 6, or 8 h. Subsequently, the cells were stained with the anti-HLA-A2 mAb to evaluate the expression of HLA-A*0201 molecules.

### 2.5. Plasmid Construction and Cell Transfection

 The mammalian expression plasmid pCI-MAGEA4, which contains the encoding sequence of MAGE-A4, was constructed as described below. Total RNA was extracted from LB1751-MEL cells using TRI_ZOL_ reagent (Invitrogen, USA). First-strand cDNA was synthesized and PCR was performed using High Fidelity PrimeScript RT-PCR Kit (TaKaRa, Dalian, China) and primers (forward, 5′-TGCCCTGACCAGAGTCATCAT-3′; reverse, 5′-ACAGAGTGAAGAATGGGCCT-3′), according to the manufacturer's instructions. The amplified products were inserted into pMD18-T plasmid (TaKaRa, Dalian, China) and the plasmid cloned into cDNA sequence of MAGE-A4 was selected and identified by restriction endonuclease digestion and sequencing. And then the encoding sequence of MAGE-A4 was amplified from the selected plasmid above and cloned into Nhe I/Mlu I sites of pCI-neo plasmid (Promega, Beijing, China) using primers 5′-TCTAGCTAG CATGTCTTCTGAGCAGAAGAGTCAGC-3′ (forward) and 5′-CCTACGACGCGTTCAGACT CCCTCTTCCTCCTCTAAC-3′ (reverse).

To establish a cell line expressing both HLA-A*0201 and MAGE-A4, MCF-7 cells were transfected with plasmid pCI-MAGEA4 using Lipofectamine 2000 (Invitrogen, USA) and then selected with G418. The expression of MAGE-A4 in the established cell line (designated MCF-7A4) was confirmed by reverse transcription-PCR and western blotting.

### 2.6. Induction of CTLs from Human PBMCs

 PBMCs were isolated from the buffy coat of heparinized whole blood samples of healthy HLA-A*0201 donors by density gradient centrifugation on the Ficoll-Paque PREMIUM (GE Healthcare Bio-Sciences AB, Uppsala, Sweden). The effector lymphocytes and dendritic cells (DCs) were prepared by our published method [29]. All donors signed written, informed consent to provide whole blood samples used in the study. Approval of the study was obtained from the relevant ethical committees and was in accordance with the Declaration of Helsinki.

### 2.7. Cytotoxicity Assay

 Three to five days after the final stimulation, the cytotoxic activity of the effector cells was evaluated by a lactate dehydrogenase release assay using Cytotox 96 Non-Radioactive Cytotoxicity Assay kit (Promega, USA) [30]. In brief, 1×10^4^ target cells (LB1751-MEL, MCF-7 or MCF-7A4) in 50 **μ**L RPMI 1640 containing 5% fetal calf serum (FCS) was placed in the wells of a 96-well round-bottom plate, then 50 **μ**L of various concentrations of effector cells was added at different effector to target (E/T) ratios (50/1, 25/1, and 12.5/1). After 4 h incubation at 37°C, the supernatant was collected to assay lactate dehydrogenase (LDH) release by OD_490_ measurement according to the manufacturer's instructions. Experiments were performed in triplicates and the percentage of lysis was calculated as % Lysis = [(experimental LDH release − effector spontaneous LDH release − target spontaneous LDH release)/(target maximum LDH release − target spontaneous LDH release)] × 100.

### 2.8. Analysis of In Vivo Immunogenicity

 HLA-A*0201/K^b^ mice were immunized with 100 **μ**g of various peptides prepared in IFA or IFA emulsion without peptide as a control. After 10 days, mice were sacrificed and splenocytes were cultured for 5 days with 10 units/mL recombinant murine interleukin-2 (rmIL-2) and **μ**g/mL peptide. And then, effector cells were counted and tested for cytotoxic activity in a cytotoxicity assay.

### 2.9. Statistical Analysis

 Statistical analyses were performed using the variance test and Student's *t*-test. A difference was considered significant at the conventional level of *P* < .05.

## 3. Results

### 3.1. Prediction of HLA-A*0201-Restricted CTL Epitopes

 The HLA-A*0201-restricted CTL epitopes of MAGE-A4 were predicted using BIMAS software. The top four ranking peptides with BIMAS scores were selected and then verified using the program SYFPEITHI. As shown in Table 1, all of the four peptides had high SYFPEITHI scores. Thus, they were selected as the candidate eptitope peptides.

### 3.2. Affinity of Candidate Epitope Peptides for HLA-A*0201 Molecule

**Figure 1:**
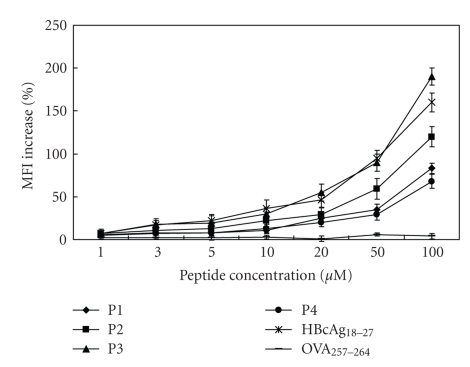
Binding affinity of peptides for HLA-A*0201 molecule. T2 cells were incubated with indicated concentrations of the peptides in serum-free RPMI 1640 medium supplied with 3 **μ**g/mL human **β**2m at 37°C for 16 h. And then the cells were stained with anti-HLA-A2 mAb and FITC-labeled goat antimouse IgG. The expression of HLA-A*0201 on T2 cells was determined with FACS Calibur flow cytometer. The peptides HBcAg_18–27_ and OVA_257–264_ were taken as positive control and negative control, respectively. Each sample was measured in three replicates and the experiment was repeated three times.

 We then evaluated the binding affinity of these candidate epitope peptides for HLA-A*0201 molecule *in vitro* using a T2-cell-peptide binding test.  Figure 1 showed that P3 had highest affinity for HLA-A*0201 molecule and P2 was lower affinity peptide, while P1 and P4 had lowest affinity. The negative control peptide did not increase the expression of HLA-A*0201 molecule on the T2 cell surface at all indicated peptide concentrations.

Because a stable peptide-MHC complex is very important for the induction of an antigen-specific CTL immune response [29, 31, 32], we further investigated the capacity of candidate epitope peptides to stabilize the HLA-A*0201 molecule (Table 2). The results indicated that P3 exhibited highest stabilization capacity (DC_50_ 8 h) and P4 was weak stabilizer of HLA-A*0201 molecule (DC_50_ 2 h). P2 had a binding affinity higher than that of P1 (Figure 1), but P2 stabilized HLA-A*0201 molecule more weakly than P1 (DC_50_ 2 h and 4–6 h, resp.). 

### 3.3. In Vitro Induction of Peptide-Specific CTLs

**Figure 2:**
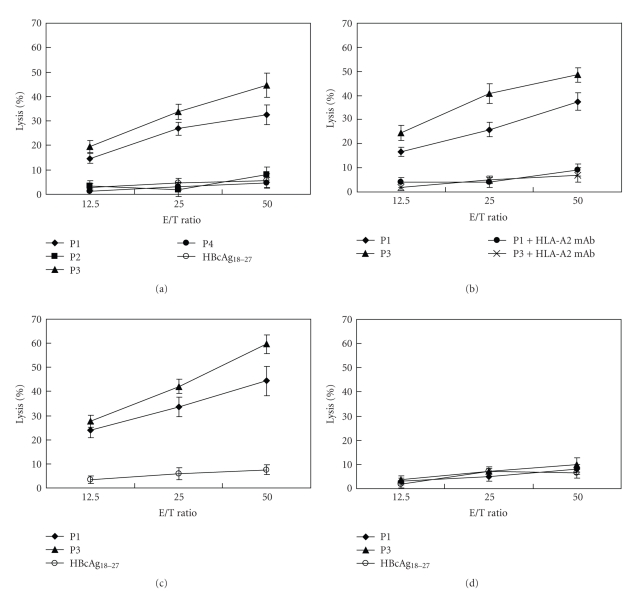
Induction of peptide-specific CTLs from human PBMCs. PBMCs from healthy HLA-A*0201 donors were first stimulated with peptide-pulsed autologous DCs and then restimulated with peptide-pulsed autologous PBMCs. After three to five days of the final stimulation, the stimulated PBMCs were used as effector cells to detect their cytotoxic activity against tumor cells at the indicated E/T ratios in a cytotoxicity assay. The irrelevant peptide HBcAg_18–27_ was taken as negative control. Each sample was measured in three replicates and the experiment was repeated three times. (a) P1, P2, P3, and P4-stimulated PBMCs mediated lysis of LB1751-MEL cells. (b) P1- and P3-stimulated PBMCs mediated lysis of LB1751-MEL cells (P1 and P3) and LB1751-MEL cells which surface HLA-A*0201 molecules were blocked with ant- HLA-A*0201 mAb (P1+HLA-A2 mAb and P3+HLA-A2 mAb). (c) P1- and P3-stimulated PBMCs mediated lysis of MCF-7A4 cells. (d) P1- and P3-stimulated PBMCs mediated lysis of MCF-7 cells.

 To study whether these candidate epitope peptides can induce the generation of peptide-specific CTLs *in vitro*, PBMCs from 3 HLA-A*0201 individuals were prepared and stimulated with peptide-pulsed autologous DCs and PBMCs successively. The cytotoxic activity of the stimulated PBMCs (effector cells) was evaluated using a cytotoxicity assay. The data from one representative donor was shown in Figure 2. The results showed that P1 and P3-stimulated PBMCs could significantly lyse the target cells LB1751-MEL. However, similar to the irrelevant peptide HBcAg_18–27_, P2- and P4-stimulated PBMCs could not lyse the target cells (Figure 2(a)). After blocking HLA-A*0201 molecules on the surface of LB1751-MEL cells with anti-HLA-A2 mAb, the lysis of LB1751-MEL cells by the effector cells was significantly abrogated (Figure 2(b)). Moreover, P1- and P3-primed effector cells could also lyse MCF-7A4 cells expressing both HLA-A*0201 and MAGE-A4 (Figure 2(c)), but not MAGE-A4-negative MCF-7 cells (Figure 2(d)). The similar results were obtained when the other two donors were tested with these peptides (data not shown).

### 3.4. In Vivo Induction of Peptide-Specific CTLs in HLA-A*0201/K^b^ Transgenic Mice

**Figure 3:**
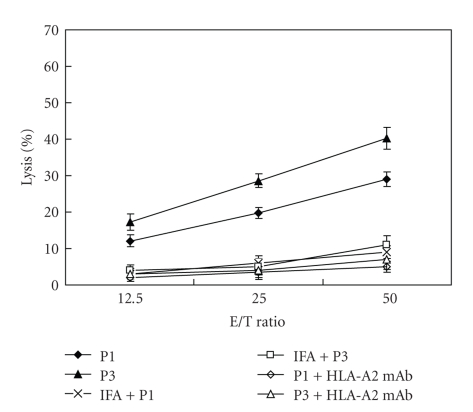
*In vivo* induction of peptide-specific CTLs. The HLA-A*0201/K^b^ transgenic mice were immunized with the peptides P1 and P3 prepared in IFA, respectively. Another group of mice were immunized with IFA without peptide as negative control. Mice were sacrificed 10 days after immunization and splenocytes were stimulated *in vitro* for 5 days with 2 **μ**g/mL peptide and 10 units/mL rmIL-2 to expand them as effector cells. A cytotoxicity assay was used to evaluate the lysis of LB1751-MEL cells by peptide-stimulated splenocytes from corresponding peptide-immunized mice (P1 and P3), P1- or P3-stimulated splenocytes from IFA-immunized mice (IFA+P1 and IFA+P3), and the lysis of LB1751-MEL cells, which surface HLA-A*0201 molecules were blocked with ant-HLA-A*0201 mAb, by peptide-immunized mice (P1+HLA-A2 mAb and P3+ HLA-A2 mAb). The experiment was repeated three times.

 Finally, we investigated whether the peptides P1 and P3 could also elicit CTL immune responses *in vivo*. The HLA-A*0201/K^b^ transgenic mice were inoculated once with the two peptides, respectively. Ten days later, the splenocytes were harvested and stimulated *in vitro *with the corresponding peptide. The cytotoxicity assay showed that the splenocytes from the P1- and P3-inoculated mice could lyse target cells LB1751-MEL, but the splenocytes from the IFA-inoculated mice could not lyse the target cells after stimulated *in vitro* with P1 or P3. When anti-HLA-A2 mAb was added, anti-HLA-A2 mAb inhibited the peptides-induced splenocytes from killing the targets (Figure 3).

## 4. Discussion

Tumor-specific immunotherapy is an appealing strategy in treating tumors. The identification of tumor-associated antigens (TAAs) and their epitopes has boosted the development of the strategy. TAAs are composed of five major groups: cancer/testis (CT) antigens (e.g., MAGE, BAGE, GAGE, NY-ESO-1, [33–35]), mutated antigens (e.g., MUM-1, p53, and beta-catenin [36, 37]), overexpressed antigens (e.g., RCAS1, Survivin, and Her2/neu [38–40]), oncofetal antigens (e.g., Immature laminin receptor and CEA [41, 42]), and differentiation or lineage antigens (e.g., tyrosinase, Melan-A/MART-1, gp100, TRP-1, and TRP-2 [43, 44]). Because CTLs play a key role in antitumor immune responses [45] and CT antigens are expressed in many tumors but not in normal tissues except testis and placenta, the identification of CTL epitopes derived from CT antigens is very important for the studies of antitumor vaccines based on defined epitope peptides.

In this study, we predicted the HLA-A*0201-restricted CTL epitopes of the tumor antigen MAGE-A4 with a combination of BIMAS and SYFPEITHI programs and selected four peptides (P1, P2, P3, and P4) as candidates based on immunogenicity score. Then we examined the binding affinity of these peptides for HLA-A*0201 and peptide/HLA-A*0201 complex stability. The results showed that P3 had both highest binding affinity for HLA-A*0201 and strongest capacity of stabilizing complex among the four peptides. P2 had intermediate binding affinity, but it exhibited weak stabilization capacity. On the other hand, P1 bound weakly to HLA-A*0201 molecules, but it could form more stable complexes with HLA-A*0201 molecules than P2 and P4. Recent studies indicate that peptide/MHC complex stability is an important parameter for distinguishing immunogenic peptides from nonimmunogenic peptides, and that a stable peptide/MHC complex may facilitate the formation of the synapses between T cells and antigen-presenting cells (APCs) and warrant the full T cell activation through sustained signaling [28, 31, 32, 46]. Our data suggested that P1 and P3 could be promising epitope candidates.

An in vitro CTL induction assay confirmed that P1 and P3 could effectively prime peptide-specific CTLs that could lyse HLA-A*0201^+^MAGE-A4^+^ target cells in an HLA-A*0201-restricted fashion, but P2- and P4-stimulated PBMCs could not lyse the target cells. At the same time, P1- and P3-stimulated PBMCs could also lyse the HLA-A*0201^+^ MCF-7A4 cells transected with *MAGE-A4* gene but not MAGE-A4-negative MCF-7 cells. The *in vivo* immunogenicity analysis found that P1 and P3 could also induce specific CTL immune responses *in vivo*. These data show that P1 and P3 are HLA-A*0201-restricted CTL epitopes and the two peptides can induce peptide-specific CTLs both *in vitro* and *in vivo* which recognize endogenously processed MAGE-A4 antigen.

Noticeably, by aligning the sequences of MAGE-A, we found that P3 (FLWGPRALA) is shared by MAGE-A1, -A4, and -A8 and that P3 is highly homologous to two peptides FLWGPRALI (shared by MAGE-A2 and -A6) and FLWGPRALV (shared by MAGE-A3 and -A12) with just one amino acid different at the carboxyl-terminus. It has been reported that the interaction between epitope peptide and MHC class I molecule mainly depends on anchor residues P2 and P9 in the nonapeptide, and the P3–P8 segment of the nonapeptide epitope contributes to the peptide/TCR interaction [28, 47–49]. The peptide FLWGPRALV is a known HLA-A*0201 restricted epitope of MAGE-A3, but it is not efficiently processed by tumor cells [50]. Therefore, we guess that P3 might be a common HLA-A*0201-restricted CTL epitope among most of MAGE-A family members and have a potential application in peptide-mediated immunotherapy, because above 80% of all tumors express at least one MAGE-A antigen. In addition, the P1 epitope (KVLEHVVRV) is shared by MAGE-A4 and MAGE-A8. The peptide seems to have homology to the HLA-A*0201-restricted CTL epitope MAGE-A1_278–286_ (KVLEYVIKV) [51], but the two epitopes exist TCR ligand sequence dissimilarity with difference at the P5, P7, and P8 residues. It remains unclear if the two epitopes KVLEHVVRV and KVLEYVIKV have the same specificity.

In conclusion, our results demonstrate that P1 (MAGE-A4_286–294_, KVLEHVVRV) and P3 (MAGE-A4_272–280_, FLWGPRALA) derived from MAGE-A4 are HLA-A*0201-restricted CTL epitopes, which can be endogenously presented to the surface of HLA-A*0201^+^MAGE-A4^+^ tumor cells. The identification of the two epitopes might provide alternative candidates for the studies of tumor-therapeutic vaccines based on defined antigenic peptides. Currently, we are investigating if P3-stimulated PBMCs can also recognize and kill the HLA-A*0201^+^ tumor cells expressing one of other members of MAGE-A family.

## Figures and Tables

**Table 1:** Predicted HLA-A*0201-restricted CTL epitopes by BIMAS and SYFPEITHI methods.

Peptide	Sequence	Position	BIMAS		SYFPEITHI	
			Score	Rank	Score	Rank
P1	KVLEHVVRV	286–294	743	1	25	4
P2	ALLEEEEGV	309–317	517	2	27	3
P3	FLWGPRALA	272–280	189	3	21	9
P4	ALPTTISFT	71–79	94	4	20	10

**Table 2:** HLA-A*0201 stabilization capacity of candidate epitope peptides.

Peptide	% MFI increase at indicated time point (h)					DC_50_^a^
	0	2	4	6	8	
P1	126.16	95.74	74.98	60.29	49.07	4–6
P2	170.07	84.13	57.09	42.34	31.19	2
P3	285.66	230.85	192.71	170.05	150.56	8
P4	98.80	48.73	30.79	21.01	18.52	2
HBcAg_18–27_	243.26	194.51	161.13	148.39	131.08	8

^(a)^Half-time of the peptide/HLA-A*0201 complex.
